# Microfluidic oxygen tolerability screening of nanocarriers for triplet fusion photon upconversion†

**DOI:** 10.1039/d2tc00156j

**Published:** 2022-02-07

**Authors:** Jussi Isokuortti, Iiro Kiiski, Tiina Sikanen, Nikita Durandin, Timo Laaksonen

**Affiliations:** Faculty of Engineering and Natural Sciences, Tampere University Tampere Finland jussi.isokuortti@tuni.fi nikita.durandin@tuni.fi; Faculty of Pharmacy, Drug Research Program, University of Helsinki Helsinki Finland

## Abstract

The full potential of triplet fusion photon upconversion (TF-UC) of providing high-energy photons locally with low-energy excitation is limited in biomedicine and life sciences by its oxygen sensitivity. This hampers the applicability of TF-UC systems in sensors, imaging, optogenetics and drug release. Despite the advances in improving the oxygen tolerability of TF-UC systems, the evaluation of oxygen tolerability is based on comparing the performance at completely deoxygenated (0% oxygen) and ambient (20–21%) conditions, leaving the physiological oxygen levels (0.3–13.5%) neglected. This oversight is not deliberate and is only the result of the lack of simple and predictable methods to obtain and maintain these physiological oxygen levels in an optical setup. Herein, we demonstrate the use of microfluidic chips made of oxygen depleting materials to study the oxygen tolerability of four different micellar nanocarriers made of FDA-approved materials with various oxygen scavenging capabilities by screening their TF-UC performance over physiological oxygen levels. All nanocarriers were capable of efficient TF-UC even in ambient conditions. However, utilizing oxygen scavengers in the oil phase of the nanocarrier improves the oxygen tolerability considerably. For example, at the mean tumour oxygen level (1.4%), nanocarriers made of surfactants and oil phase both capable of oxygen scavenging retained remarkably 80% of their TF-UC emission. This microfluidic concept enables faster, simpler and more realistic evaluation of, not only TF-UC, but any micro or nanoscale oxygen-sensitive system and facilitates their development and implementation in biomedical and life science applications.

## Abstract 2

A new microfluidic approach was used to screen the oxygen tolerability of photon upconverting nanocarriers and to quantify their performance at oxygen levels ranging from severely hypoxic tumours to healthy peripheral tissue.

## Graphical abstract

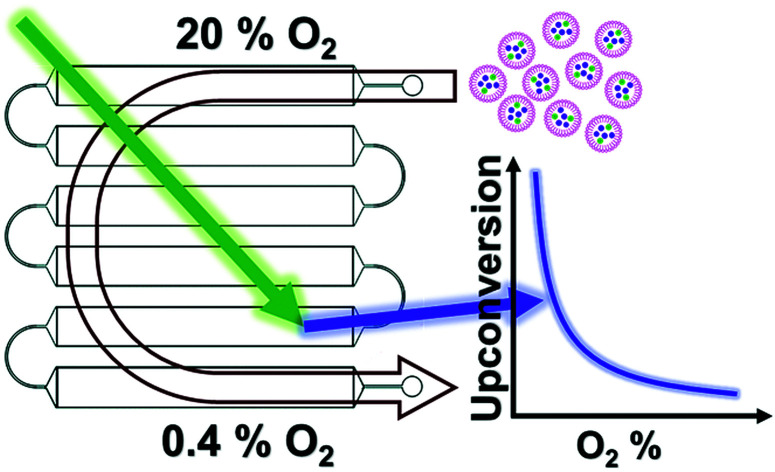

## Introduction

**Fig. 1:**
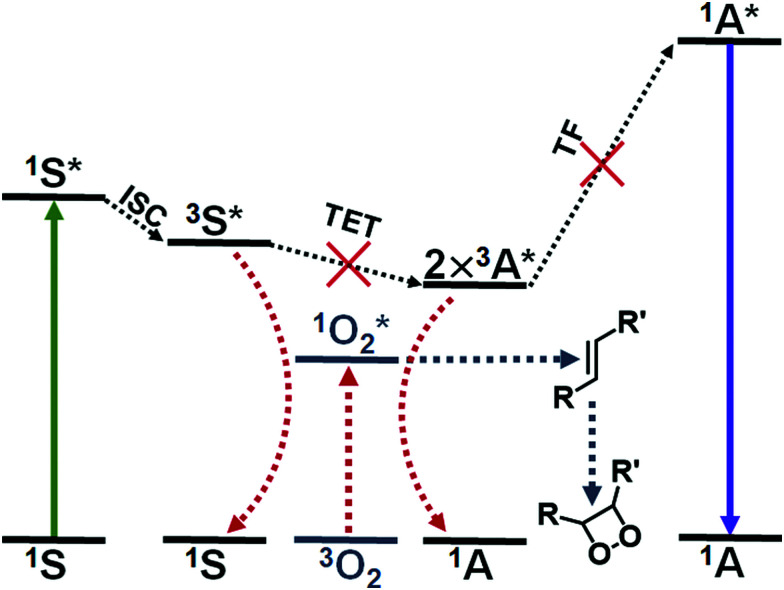
The process of triplet fusion upconversion (TF-UC): the sensitizer (S) absorbs a low-energy photon (green arrow) and undergoes intersystem crossing (ISC) to yield an excited triplet state. The energy of the excited triplet state is transferred to the acceptor molecule (triplet energy transfer, TET). Two excited triplet acceptors can then undergo triplet fusion (TF), which results in one excited singlet acceptor that fluoresces an upconverted photon. Molecular oxygen can quench the excited triplet state of either the sensitizer or the acceptor, which yields singlet oxygen and intercepts the UC process. Unsaturated compounds in the nanocarrier can react with singlet oxygen, for example, by 1,2-addition. These oxygen scavengers protect the dye molecules from oxidation and deplete oxygen from the system *in situ*, allowing the UC process to proceed.

Triplet fusion, also known as triplet–triplet annihilation, photon upconversion (TF-UC) is a process that combines the energy of two longer wavelength photons to a high-energy excited state that results in emission of one photon with shorter wavelength than the excitation (see Fig. 1). TF-UC systems have advanced greatly in the past 20 years in terms of efficiency and implementation from solution studies to nanoscale systems and solid state devices,^1–4^ which has enabled the application of TF-UC in such fields as photovoltaics, photocatalysis, sensing, bioimaging, optogenetics and phototherapy.^5–9^ The capability of generating blue or UV light *in situ via* lower energy excitation with less phototoxicity and better tissue penetration is especially valuable for the life science and biomedical applications. Despite the recent advances in the field, the severe oxygen sensitivity of TF-UC is still a major limitation to its wider use, especially in biological media such as cells and tissues, and remains to be solved.^10,11^

The issue of molecular oxygen is intrinsic to TF-UC, since it is based on excited triplet states that are quenched by molecular oxygen to yield singlet oxygen. The generation of singlet oxygen competes with not one but two steps of TF-UC, illustrated in Fig. 1, reducing the quantum yield of upconversion (QY) and increasing the excitation power density threshold (*I*_th_) of efficient UC. Singlet oxygen is also a powerful oxidant that can damage the dye molecules of the UC system or its immediate surroundings. This naturally leads to degradation of the UC system^12^ and toxicity to cells and tissue through the photodynamic effect that can be either beneficial in the case of photodynamic therapy or harmful in the case of, for example, bioimaging.^13^ Thus, the effects of molecular oxygen, when unmitigated, can lead to no upconversion and even loss of the UC system.

Several approaches have been developed to improve the oxygen tolerability of TF-UC systems in aqueous media and to enable their use *in vitro* and *in vivo*. These strategies have been outlined in the excellent reviews by Askes & Bonnet^10^ and Baluschev *et al.*^11^ Arguably, the most straightforward method of achieving TF-UC in aqueous media and in the presence of oxygen is to utilize self-assembling materials such as micelles with oxygen scavengers, since no chemical modification of the commercially available and biocompatible materials is required and the fabrication of these materials is simple. Singlet oxygen scavengers are molecules or moieties that can react with singlet oxygen (see Fig. 1) and serve two purposes in the UC system: protection of the dye molecules from oxidation and depletion of oxygen *in situ*.

Conventionally, TF-UC studies have been performed in meticulously deoxygenated media. As the research focus has been shifting from fundamental studies to the applications of TF-UC, many studies have investigated the performance of the UC system at ambient oxygen levels (20–21%) and compared the results to those achieved at anoxia (0%). This approach, however, leaves a wide range of, especially biomedically relevant, oxygen levels unexplored. The oxygen levels in human tissue range from 13.5% in lung alveoli to as low as 0.3% in severely hypoxic tumours.^14^ Thus, the performance of the TF-UC systems at these physiological oxygen levels are typically only speculated on and no realistic evaluation and screening of the systems cannot be performed prior to complicated *in vitro* and *in vivo* studies.

The neglect of TF-UC studies at the relevant physiological oxygen levels is not deliberate. Maintaining a physiological oxygen level in an optical measurement setup is not trivial and requires, for example, complicated instruments to regulate gas flow and composition. Fortunately, the recent progress in microfluidic technologies has facilitated precise control of oxygen levels in biological experiments and implementation of spatiotemporal oxygen gradients at the microscale.^15^ A particularly simple approach to control the oxygen level on microfluidic chips is to exploit the inherent oxygen scavenging ability of off-stoichiometric thiol–enes (OSTE).^16^ OSTE polymers are a new class of custom microfabrication materials, which enable not only rapid replica molding of microfluidic devices, but also controlled oxygen depletion on these devices *via* rapid reactions between the thiol moieties and dissolved molecular oxygen.^17^ In a flow-through setting, the oxygen depletion rate is dependent on the bulk composition, the surface-to-volume ratio of the microchannel, and the flow rate (residence time) of the sample solution.^16,17^ The high optical clarity and transparency of OSTE also enables optical measurements, thus making this concept well-feasible for the screening of oxygen tolerability of nano- and microscale TF-UC systems in a facile and rapid manner.

**Chart 1:**
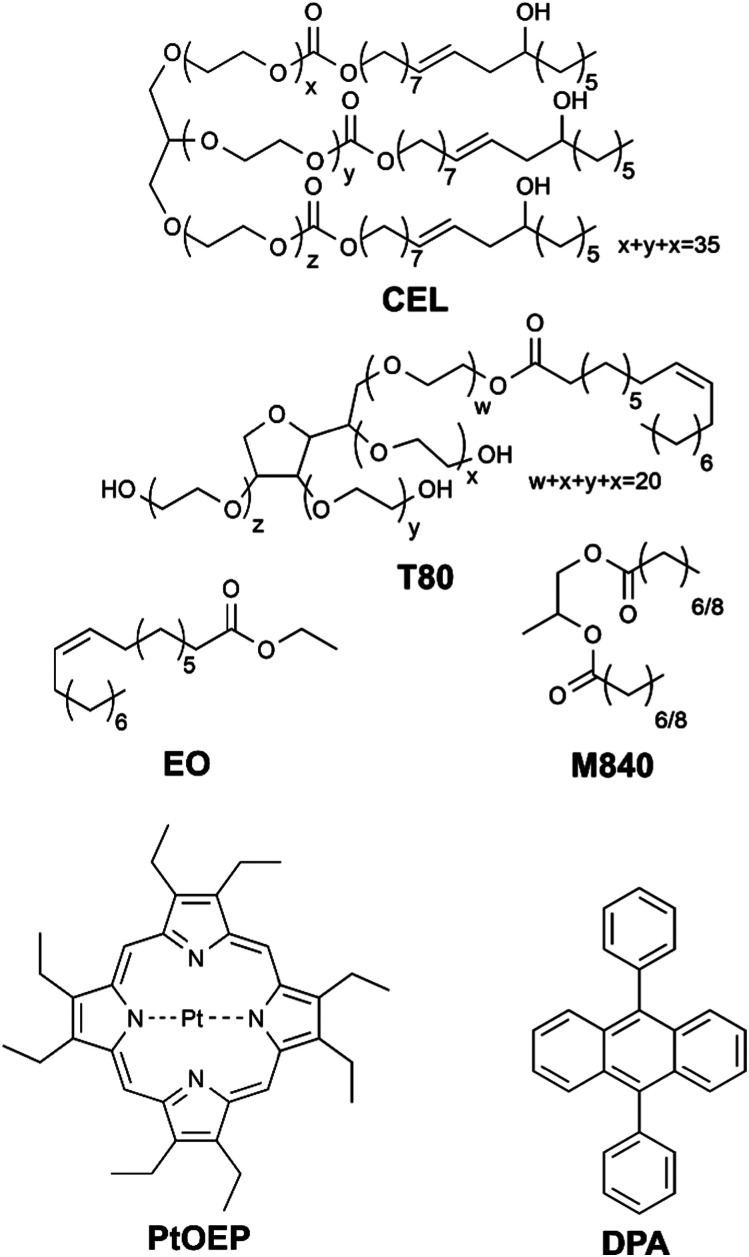
Surfactants (CEL and T80), oils (EO and M840), sensitizer (PtOEP) and acceptor (DPA) used in this study. The presence of unsaturated bonds in the CEL, T80 and EO structures enable their use as oxygen scavengers, whereas the saturated M840 has no expected oxygen scavenging abilities.

In this study we have formulated four different micellar nanocarriers for TF-UC using FDA-approved Cremophor® EL (CEL) and Tween® 80 (T80) as surfactants and ethyl oleate (EO) and Miglyol® 840 (M840) as oil phases incorporated in the hydrophobic core of the micelles along with the well-documented TF-UC pair^18,19^ of sensitizer platinum(ii) octaethylporphyrin (PtOEP) and acceptor 9,10-diphenylanthracene (DPA). The structures of these compounds are shown in Chart 1. The unsaturated bonds of CEL, T80 and EO allow them to react with singlet oxygen^20^ and can therefore operate as oxygen scavengers, whereas M840 contains only saturated bonds and cannot scavenge singlet oxygen. Due to the unsaturated bonds in the surfactants, we expect all four formulations to be able to deplete oxygen *in situ* and perform upconversion in presence of molecular oxygen. However, we also anticipate that utilizing unsaturated and singlet oxygen scavenging EO as the oil phase will improve the oxygen tolerability considerably compared to the saturated M840 oil phase.

First, we evaluate the oxygen tolerability of these green-to-violet upconverting nanocarriers with the conventional method by determining their performance at 0% oxygen and comparing the results to those achieved at ambient (21% oxygen). After these initial studies, we use the oxygen-depleting microchips to study the upconversion performance of nanocarriers over a range of oxygen concentrations from 0.4% to 5.5% to determine how these physiological oxygen levels affect the TF-UC efficiency. This information is paramount when, for example, designing nanocarrier formulations for cancer therapy or bioimaging.

## Experimental section

### Sample preparation

All chemicals except M840 were purchased from Sigma Aldrich (Merck KGaA). M840 was received as a free sample from IOI Oleo GmbH. The micellar upconversion systems were prepared as follows: 80 mg of either T80 or CEL was added to a 25 mL round bottom flask. In case of Tween 80, either 8.4 mg of M840 or 10.4 mg of EO and in case of CEL, either 11.2 mg of M840 or 14.3 mg of EO was added. 1 mg of DPA and 0.1 mg of PtOEP as dichloromethane solutions were added to flask and stirred until homogenous mixture was yielded. Dichloromethane was then removed with a rotary evaporator operated at 50 mbar and room temperature for 30 minutes. The viscous residue was then taken up by adding 3 mL of deionized water and stirring for 30 minutes. This resulted in a clear pink dispersion with total [PtOEP] = 50 μM and [DPA] = 1 mM. The size and size distribution of the micellar nanocarriers were determined with dynamic light scattering using a Zetasizer Nano (Malvern Panalytical Ltd) instrument at room temperature. Absorption spectra were measured with a UV-3600 (Shimadzu Corporation, Japan) spectrophotometer.

### Microchip setup

The microfluidic chips were fabricated as previously described^17^ by mixing the tetrafunctional thiol monomer (pentaerythritol tetrakis(3-mercaptopropionate), Bruno Bock, Marschacht, Germany) and the trifunctional ‘ene’ monomer (triallyl-1,3,5-triazine-2,4,6(1*H*,3*H*,5*H*)-trione, Sigma Aldrich) in a ratio that yielded an 25% excess of free thiol groups in the polymer bulk. The chip design comprised six serially interconnected micropillar channels (4 × 30 × 0.2 mm^3^, width × length × height), each incorporating *ca.* 14 400 micropillars (diameter 50 μm). The sample dispersions were fed into the chip with a programmable Cole-Parmer 78-9100C single-syringe infusion pump (Cole-Parmer Instrument Company, LLC) and a 1 mL gas-tight HPLC syringe (Hamilton Company). The oxygen level was controlled by adjusting the flow rate (2.5–20 μL min^−1^) and measured at the end of the oxygen-depleting 6-channel array by using a Piccolo2 oxygen meter (PyroScience GmbH) and OXNANO nanoprobes (PyroScience GmbH) to establish a correlation between the flow rate and the oxygen concentration (%).

### Upconversion measurements

Upconversion measurements were performed by using a Verdi V6 532 nm second harmonic Nd:YAG laser (Coherent Inc.) and an Avaspec 2048 spectrometer (Avantes BV) with a detection range of 400–800 nm. Laser power was modulated by a set of neutral density filters (Edmund Optics Ltd). The cuvettes used were 1 cm^2^ SOG9 fluorescence cuvettes with a rubber septum (anoxia measurements) or without a cap (ambient). Anoxia was induced by adding 30 mM of Na_2_SO_3_ to the cuvette.

The laser beam was focused to a 1.6 mm diameter spot on the chip or a 0.8 mm spot on the cuvette. The spot size was measured with a LBP2-HR-VIS2 beam profiler (Newport Corp.). Emission light was collected from the microfluidic chip at a 45 degree angle relative to the excitation beam with a single lens to a 1 mm diameter light guide coupled to the spectrometer. Emission light was collected from the cuvette by butt-coupling the light guide to the cuvette wall at a 90 degree angle to the laser beam. Upconversion quantum yields were determined by using rhodamine 6G as a reference without using the multiplication factor of 2.

## Results and discussion

### Micellar nanocarriers for TF-UC

The nanocarriers were based on micelles with incorporated high boiling point solvent (oil) in the hydrophobic core. The oil phase, when compared to micelles without one, can significantly improve the TF-UC performance by reducing dye aggregation^21^ and offers means for adding oxygen scavengers to the formulation. Since the primary aim of this study was to focus on developing and characterizing TF-UC systems for biomedicine and life sciences, the surfactants and oils used in this work were selected from clinically approved ingredients. Another selection criterion was the capability of the nanocarriers to solubilize sufficiently high dye concentrations needed to yield efficient UC, while maintaining monodisperse size distribution and stability. As a result of the optimization, it was observed that, for example, micelles made of Tween® 20 or Pluronic® F127 were not able to incorporate high enough oil and dye load without phase separation. Instead, the CEL and T80 micellar nanocarriers were highly monodisperse (polydispersity index <0.1) with hydrodynamic diameters between 10 and 20 nm (Table S1, ESI†) and could hold high enough oil and dye loads for efficient TF-UC. Compared with T80, CEL was able to incorporate higher mass percentage of both oils tested (EO and M840), with oil loadings of 12–15 wt% (CEL) *versus* 10–12 wt% (T80). The chosen dye load (approximately 1 wt% for DPA and 0.1 wt% for PtOEP) was the highest that the T80 + M840 nanocarriers were able to solubilize and was kept constant for all formulations to maintain equal optical densities for the UC measurements.

### Characterization of the UC nanocarriers in deoxygenated medium

**Fig. 2:**
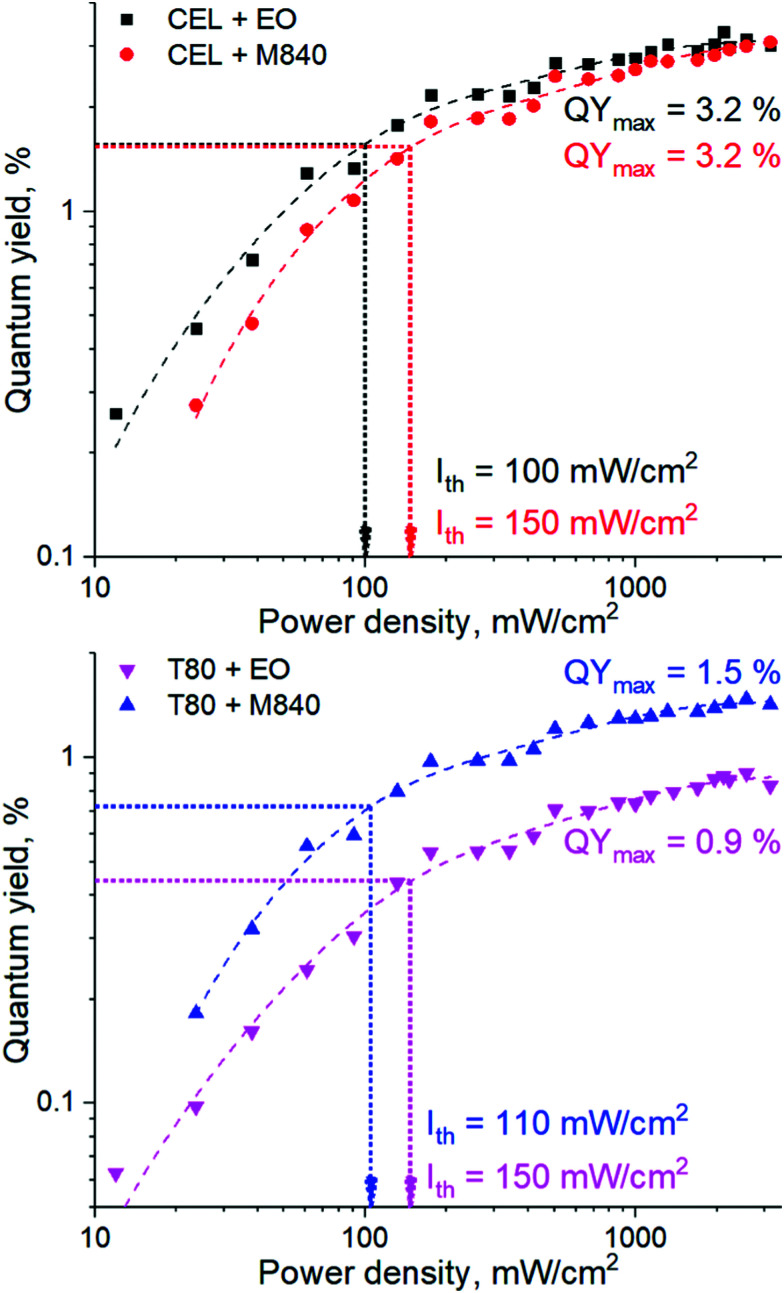
Quantum yield of upconversion (QY) of CEL (top) and T80 (bottom) nanocarriers as a function of *I*_exc_ in deoxygenated water. The dashed lines represent two-exponential fits on the data. The *I*_th_ values were determined as the *I*_exc_ that yielded half of the maximum QY for each system and are indicated with the dotted arrows.

The performance of the UC nanocarriers was initially studied by determining the quantum yield (QY) of upconversion under a range of excitation power densities (*I*_exc_) in completely deoxygenated water. Deoxygenation was performed by adding an excess of a common oxygen scavenger, Na_2_SO_3_ (30 mM), to the solution. The quantum yields of each system under varied excitation power densities along with corresponding power density thresholds (*I*_th_) are shown in Fig. 2. The QYs are reported without the multiplication factor of 2 (*i.e.* maximum theoretical QY is 50% due to TF-UC being a two-photon process). *I*_th_ was determined for each nanocarrier formulation by finding the power density that yields half of the maximum QY.^18,22^ The emission spectra of all nanocarriers are shown in Fig. S3 (ESI†).

Both surfactant and oil phase of the nanocarrier had a strong effect on the UC performance. The higher QYs (3.2%) obtained with the CEL nanocarriers are likely the result of a higher dye loading per nanocarrier. Interestingly, *I*_th_ seems to be determined mostly by the oil phase. This can be attributed to the viscosity of the oil and thus the fluidity of the micellar core. Since M840 is more viscous than EO (11 cP^23^*versus* 6 cP^24^ at room temperature, respectively), the triplet energy transfer and triplet fusion steps that require molecular collisions may occur more efficiently in EO, thus resulting in lower *I*_th_ for EO containing nanocarriers. This is evident also from the emission spectra (see Fig. S3, ESI†) of the nanocarriers as the M840 containing nanocarriers exhibit less quenching of PtOEP phosphorescence than the corresponding EO containing nanocarriers. Despite the range of obtained QYs and *I*_th_, all nanocarriers are capable of efficient TF-UC in deoxygenated conditions.

### Self-deoxygenation properties of the nanocarriers

**Fig. 3:**
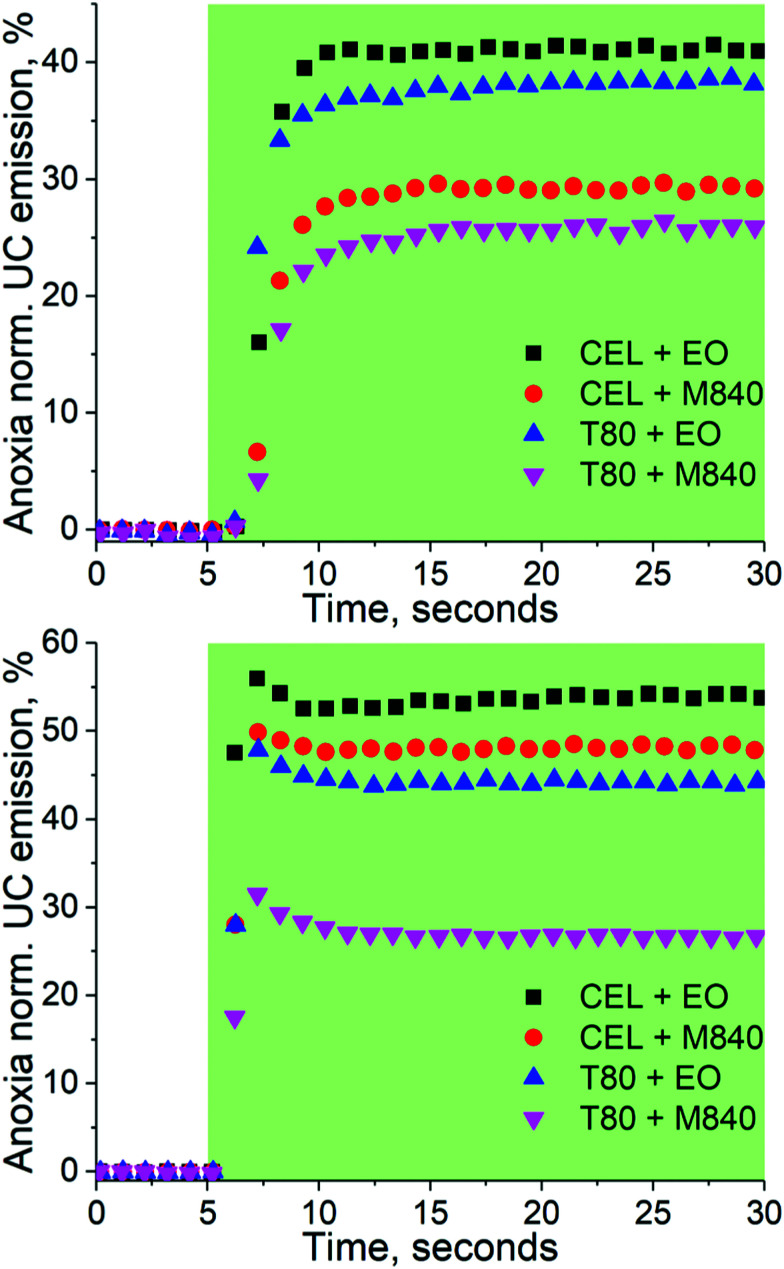
Deoxygenation curves of the nanocarriers under 260 (top) and 1000 mW cm^−2^ (bottom) excitation. The green sections indicate the time under excitation, *i.e.* the excitation was started after the first 5 seconds. The data is normalized by the emission intensity obtained at anoxic conditions. The emission spectra recorded at the 30 s mark are shown in Fig. S4 and S5 (ESI†).

The capabilities of the nanocarriers to deoxygenate *in situ* under excitation and thus enabling TF-UC in ambient conditions was studied by exciting the samples in a cuvette without any prior removal of oxygen under two different *I*_exc_, 260 and 1000 mW cm^−2^, and monitoring the UC emission intensity over time. The deoxygenation capability is evaluated by comparing the UC emission intensity at ambient to the UC emission intensity obtained at anoxia. The resulting transient emission curves are shown in Fig. 3.

With the lower *I*_exc_ of 260 mW cm^−2^, the formulations containing the oxygen scavenging EO expectedly outperform M840 containing nanocarriers. When *I*_exc_ is increased to 1000 mW cm^−2^, the contribution of the oxygen scavenging oil phase to the UC performance diminishes as the CEL + EO, CEL + M840 and T80 + EO perform similarly. This can perhaps be explained with the UC process being overall more efficient in the CEL nanocarriers and thus it can compete with the generation of singlet oxygen at higher *I*_exc_. The difference in UC performance at higher and lower *I*_ex_ also highlights the importance of choosing *I*_exc_ relevant to the application, since exaggerated *I*_exc_ may lead to overestimation of the self-deoxygenation capabilities when *I*_exc_ is limited, for example due to tissue penetration. At lower *I*_exc_ the utilization of oxygen scavengers in the oil phase is clearly beneficial. It should be also noted, that without any oxygen scavenging moieties, whether in the surfactant or in the oil phase, no UC could be detected.

We also studied the stability of our UC nanocarriers in ambient conditions by monitoring their UC emission under 1000 mW cm^−2^ excitation for one hour (see Fig. S7, ESI†) and measuring their absorption spectra before and after (see Fig. S8, ESI†). All nanocarrier formulations were able to maintain UC during one hour of continuous excitation. No photobleaching of PtOEP was observed, while DPA absorbance decreased approximately 4 and 10% in EO and M840 containing micelles, respectively, indicating that oxygen scavenging oil phase is also beneficial for the long-term photostability of the nanocarriers. These stability studies also reveal that the differences observed in the UC performance in ambient or anoxic are predominantly a result of oxygen quenching the excited triplet states rather than, for example, photooxidation of the dyes.

### Upconversion studies under varied oxygen levels

**Fig. 4:**
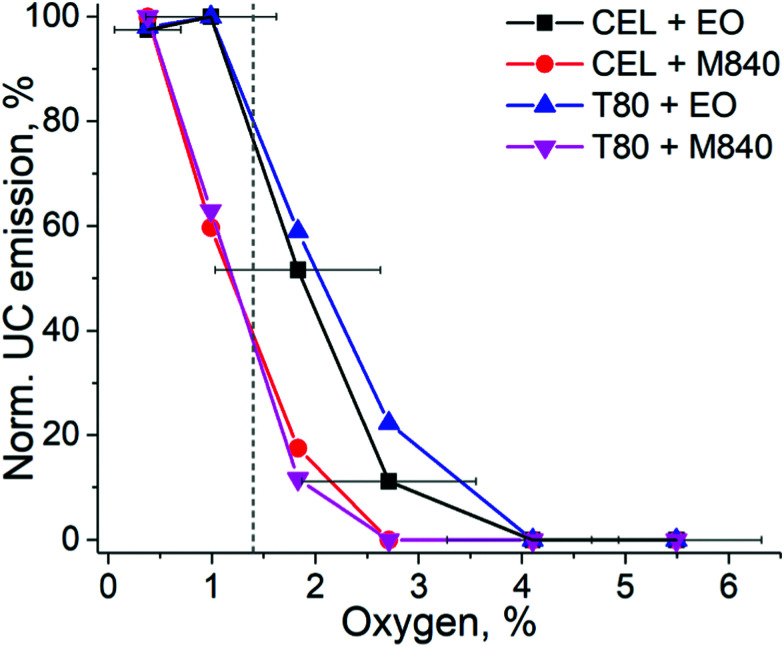
UC emission intensities of each nanocarrier obtained by varying the oxygen concentration in the microfluidic chip under 140 mW cm^−2^ excitation. The dashed grey line indicates the mean oxygen concentration (1.4%) in human tumours. The data is normalized by the highest UC intensity of each composition measured from the chip. The error bars indicate the standard deviation of oxygen % in the chip (*n* = 3). The oxygen levels at the used flow rates are shown in Table S2 (ESI†).

To evaluate the UC performance of the nanocarriers at physiologically relevant oxygen levels, we used the oxygen-depleting microfluidic chips.^17^ The chip is fabricated from an oxygen-depleting OSTE formulation and comprises a long micropillar-filled channel that gradually scavenges oxygen from the in-flowing liquid. The on-chip oxygen level can be controlled simply by adjusting the liquid residence time inside the chip with flow rate: the highest flow rate of 20 μL min^−1^ resulted in approximately 5.5% oxygen concentration at the chip outlet (20–21% being the ambient level of oxygen) while the lowest flow rate of 2.5 μL min^−1^ resulted in approximately 0.4% oxygen concentration (see Table S2, ESI†). This range covers the oxygen levels encountered in severely hypoxic tumours (0.3%) to the average oxygen level in healthy peripheral tissue (6%).^14^ The normalized UC emission intensities of each nanocarrier formulation in this range of oxygen levels are shown in Fig. 4.

As expected, based on the molecular structure, the EO-containing nanocarriers are capable of sustaining UC at higher oxygen levels than M840-containing ones. EO-loaded nanocarriers can maintain the initial maximum UC emission until the oxygen level reaches 1%, while M840-loaded nanocarriers exhibit a decrease to 60% at 1% oxygen. With EO, UC emission is detected even at 2.7% oxygen. When the changes in normalized UC emission are compared, the higher density of double bonds in CEL (three double bonds per surfactant molecule) does not make the CEL nanocarriers more tolerant towards oxygen than the T80 nanocarriers (one double bond per surfactant molecule). This was also the case when the deoxygenation properties were studied at ambient oxygen and under the lower *I*_exc_ of 260 mW cm^−2^. It should also be noted that the QYs of the nanocarriers differ and thus the absolute UC emission intensities vary between the nanocarriers at different oxygen levels (see Fig. S10, ESI†). For example, at the mean tumour oxygen level of 1.4%, CEL + EO nanocarriers exhibited approx. 1.4% QY, while only approx. 0.2% QY was obtained for T80 + M840 nanocarriers.

Since cancer therapies and imaging are a significant application field for UC systems, we have used the mean tumour oxygen level of 1.4%^14^ as the benchmark for our nanocarriers. At this oxygen level, the EO-containing nanocarriers retain approximately 80% of their UC emission, whereas M840-containing nanocarriers have their UC emission reduced to approximately 40%. When approaching healthy peripheral tissue oxygen levels (6%), no upconversion was detected regardless of the formulation. This comparison reveals the substantial differences between nanocarrier formulations at physiologically relevant oxygen concentrations and demonstrates that the EO-containing nanocarriers can be useful for cancer related applications. Such findings could not have been made nor confirmed without the microfluidic setup. As such, the microfluidic approach thereby improves both the versatility of the assay designs and reliability of the results compared with current standard protocols. Typically, the UC performance is only determined at complete anoxia or at ambient level of oxygen, leaving the performance at relevant physiological oxygen levels undetermined and speculative.

Naturally, the microfluidic approach can also be expanded to other applications where the photochemical performance is dependent on molecular oxygen, such as photodynamic therapy,^25^ light-triggered drug release by photooxidation,^26^ and some photocatalytic processes^27^ and even to their high-throughput experimentation. The ability to accurately and rapidly determine the UC performance under a range of oxygen levels can for example facilitate the design of more selective imaging agents and safer cancer therapies due to the difference in oxygen levels in healthy and cancerous tissue. At best, this could enable the design of UC-based drug release systems that perform only in the tumour and have no response to light in a healthy tissue.

## Conclusions

We have formulated four micellar nanocarriers with varying oxygen scavenging capabilities made of FDA-approved surfactants and oils for green-to-violet triplet fusion upconversion and studied their upconversion performance under a range of oxygen levels. In completely deoxygenated conditions, the maximum quantum yields of upconversion were 0.9–3.2% (out of 50% theoretical maximum) with power density thresholds of 100–150 mW cm^−2^. Each formulation was capable of upconversion even in ambient conditions, however, utilizing an oxygen scavenger as the oil phase improves the oxygen tolerability considerably. Interestingly, the number of oxygen scavenging double bonds (one *versus* three in T80 and CEL, respectively) in the surfactant molecule seems to have little to no effect on the oxygen tolerability. To study the upconversion performance at physiological oxygen levels – an aspect that has been much overlooked in previous studies – we have utilized oxygen-depleting microfluidic chips that allow precise control of the oxygen levels in a facile and rapid manner. The oxygen-scavenging oil phase improved the oxygen tolerability of the nanocarriers also at these intermediate oxygen levels: their TF-UC (relative) emission at 100% remained unchanged until 1% oxygen and at the mean tumour oxygen level of 1.4%, oxygen scavenger loaded nanocarriers maintained their UC emission at 80% of the maximum. Nanocarriers without an oxygen-scavenging oil phase had considerably poorer oxygen tolerability as TF-UC emission decreased to 60% at 1% oxygen and 40% at 1.4% oxygen. Overall, the microfluidic approach allows more realistic screening of oxygen-sensitive nano- and microscale systems prior to more complicated *in vitro* and *in vivo* studies and can even enable their high-throughput experimentation. This concept will thus help develop, for example, more accurate sensors, brighter imaging probes and more effective and selective cancer therapies.

## Conflicts of interest

There are no conflicts to declare.

## Supplementary Material

TC-010-D2TC00156J-s001
