# Supplementary material for: Microfluidic oxygen tolerability screening of nanocarriers for triplet fusion photon upconversion
Source: J Mater Chem C Mater. 2022 Feb 7;10(12):4871–7. doi: 10.1039/d2tc00156j (PMC8944590; doi:10.1039/d2tc00156j)
Supplement: TC-010-D2TC00156J-s001 [file TC-010-D2TC00156J-s001.pdf]

Electronic Supplementary Material (ESI) for Journal of Materials Chemistry C.
This journal is © The Royal Society of Chemistry 2022

Supplementary information

Microfluidic oxygen tolerability screening of nanocarriers for triplet fusion photon upconversion

Jussi Isokuortti*[a], Iiro Kiiski [b], Tiina Sikanen[b], Nikita Durandin*[a] and Timo Laaksonen[a,b]

[a] Faculty of Engineering and Natural Sciences, Tampere University, Tampere, Finland.
Email: jussi.isokuortti@tuni.fi, nikita.durandin@tuni.fi

[b] Faculty of Pharmacy, Drug Research Program, University of Helsinki, Helsinki, Finland.

* Corresponding authors.

Contents

Size and polydispersity index of the nanocarriers 1
On-chip oxygen levels 1
Upconversion measurements 1
Determination of upconversion quantum yields 2
Stability of nanocarriers under excitation in ambient conditions 6
Upconversion emission measurements from the microfluidic chip 7
Upconversion emission intensities at varied oxygen levels normalized by quantum yields 9
References 10

Size and polydispersity index of the nanocarriers

The size and polydispersity index of the nanocarriers was determined with dynamic laser scattering at 22 °C in deionized water. The size (diameter) and polydispersity indexes of the nanocarriers are shown in Table S1.

Table S1 Size and polydispersity index of the nanocarriers in water.

	Size, nm	Polydispersity index
CEL + EO	17.3	0.03
CEL + M840	16.4	0.03
T80 + EO	12.4	0.07
T80 + M840	14.3	0.08

On-chip oxygen levels

The oxygen levels inside the microfluidic chip were determined by using an optical oxygen sensor (Piccolo2, Pyro Science, Aachen, Germany) with nanoprobes (OXNANO, Pyro Science, Aachen, Germany) dispersed in water. The oxygen levels at the measurement spot at the flow rates used in the study are shown in Table S2.

Table S2 Mean oxygen levels (n = 3) and their standard deviation (in parenthesis).

Flow rate, µl/min	Oxygen level, %
20	5.5 (0.8)
15	4.1 (0.8)
10	2.7 (0.8)
7.5	1.8 (0.8)
5	1.0 (0.6)
2.5	0.4 (0.3)

Upconversion measurements

The schemes of the upconversion measurements from cuvette and from microfluidic chips are shown in the Figures S1 and S2.

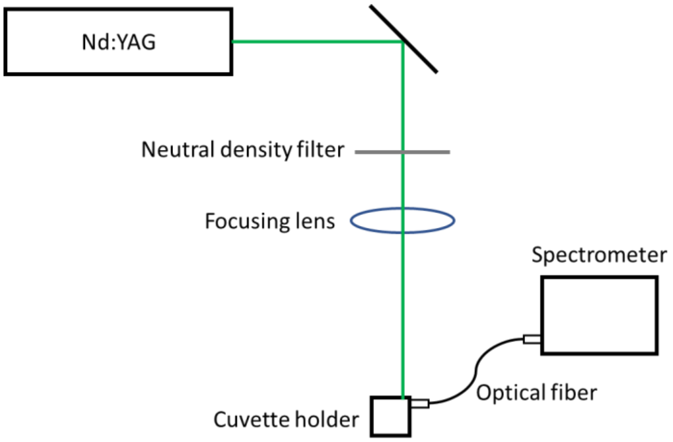

Figure S1 Scheme of the cuvette measurements.

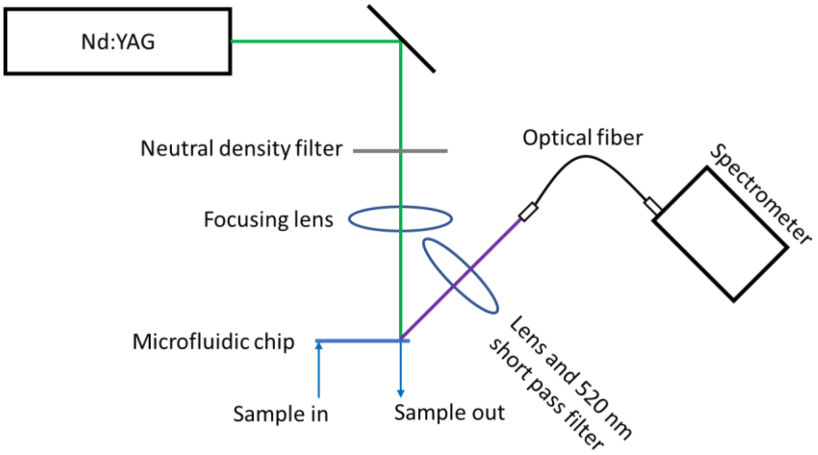

Figure S2 Scheme of the microfluidic chip measurements, the violet line depicts the upconversion emission from the chip.

Determination of upconversion quantum yields

The upconversion quantum yields (QY) at 0 % oxygen (by using 30 mM of Na_2SO_3) were determined by integrating the emission spectra of the nanocarriers from 400 to 520 nm and using dilute (maximum absorbance less than 0.1) Rhodamine 6G in ethanol as a reference (95 % fluorescence quantum yield[1], emission spectrum integrated from 525 to 800 nm). The quantum yields were then calculated using the following equation[2]:

$$QY=\Phi_{std}\left(\frac{A_{std}}{A}\right)\left(\frac{I_{UC}}{I_{std}}\right)\left(\frac{\eta}{\eta_{std}}\right)^2 \qquad (S1)$$

where Φ_{std} is the quantum yield of the reference, A is the absorbance at the excitation wavelength, I is the integrated emission intensity and η is the refractive index of the medium

(1.33 for water and 1.36 for ethanol). Note that the quantum yields were determined without the multiplication factor of 2.

The 532 nm excited emission spectra of the nanocarriers are shown in Figure S3.

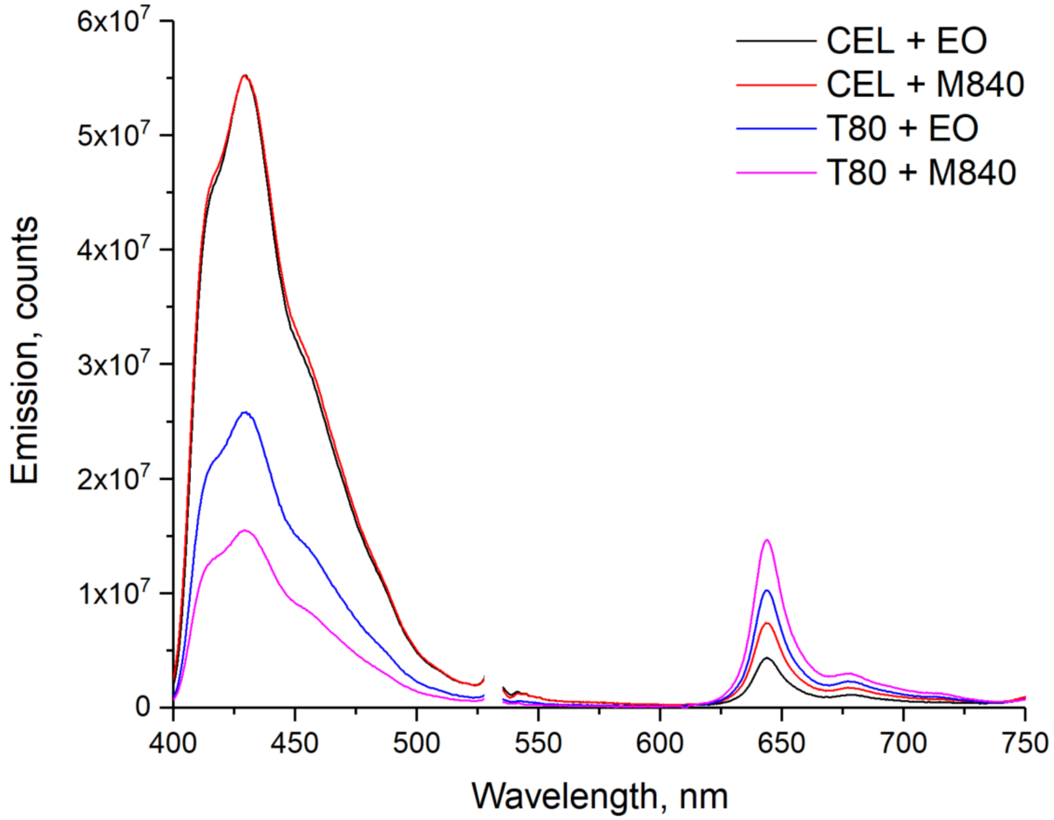

Figure S3 Emission spectra of the nanocarriers at anoxic conditions under 3100 mW/cm^2 excitation, which yielded the maximum quantum yield of upconversion. Scattering from the 532 nm excitation is excluded for clarity.

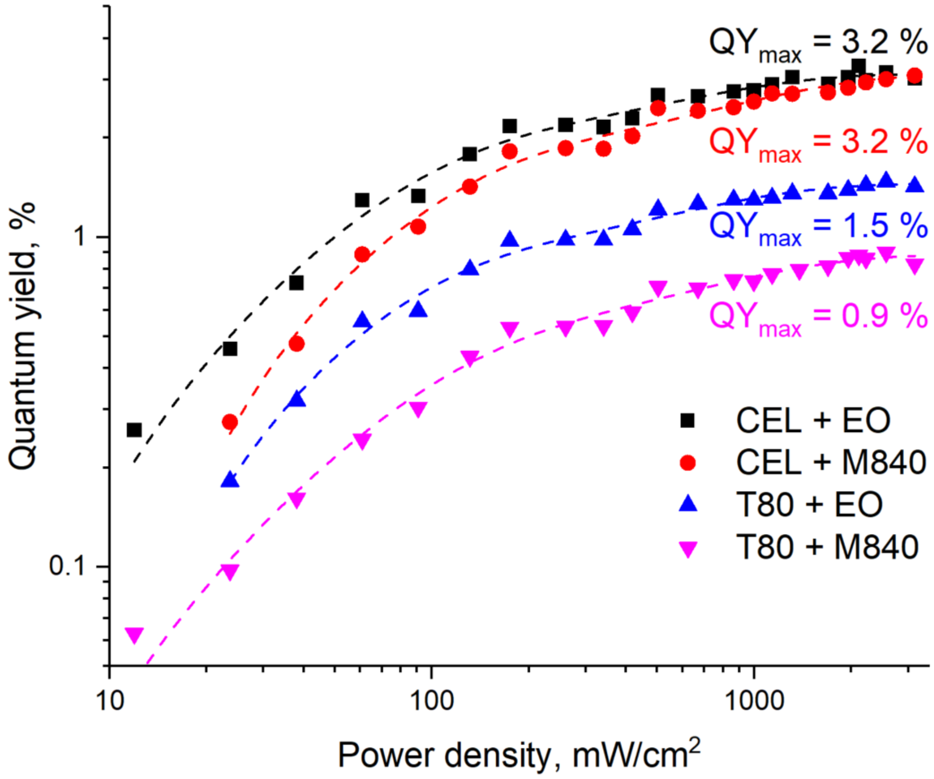

Figure S4 Quantum yield of upconversion under varied excitation power density of each nanocarrier system.

Upconversion performance at ambient conditions was determined by comapring the emission spectra measured at ambient conditions with the emission spectra obtained at anoxic conditions. The emission spectra (at anoxic and ambient conditions) obtained at 1000 mW/cm^2 and 260 mW/cm^2 excitation are shown in Figures S4 and S5.

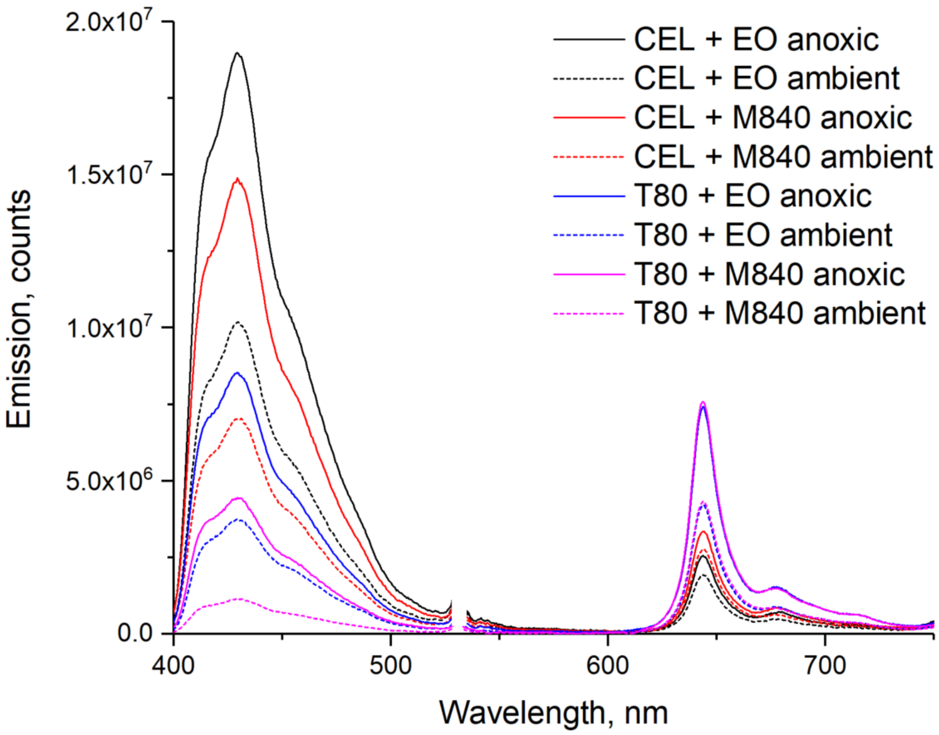

Figure S5. Emission spectra obtained at 1000 mW/cm^2 excitation at both anoxic and ambient conditions.

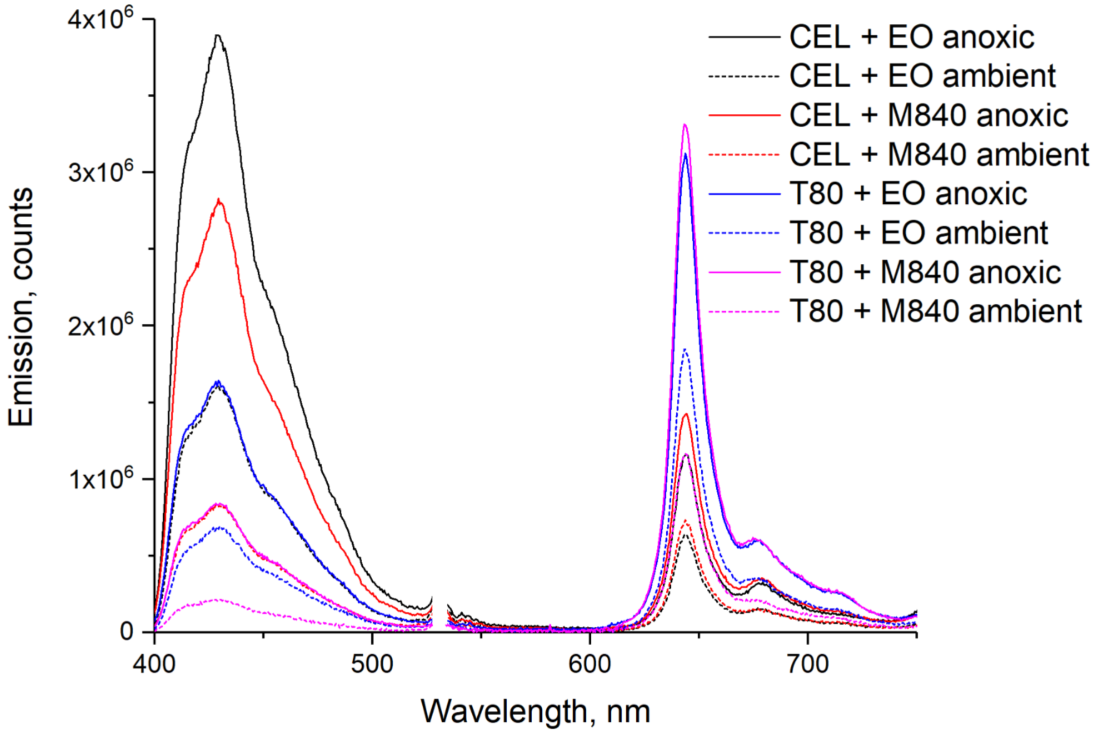

Figure S6. Emission spectra obtained at 260 mW/cm^2 excitation at both anoxic and ambient conditions.

Stability of nanocarriers under excitation in ambient conditions

The UC emission was monitored over 1 hour period (see Fig. S4) in ambient conditions (the cuvette was uncapped) under 1000 mW/cm^2 excitation. The results were normalized by the UC emission yielded by the nanocarriers at anoxic conditions. To study the photobleaching of the samples, absorption spectra were measured before and after 1 hour of excitation (see Fig. S5, shown for T80 + EO and T80 + CEL, diluted 10 times). No changes in the PtOEP Q band absorbance were noticed with any samples and thus the slight decrease in absorbance between 325 and 425 nm in the samples was attributed to the photobleaching or photooxidation of DPA. CEL + EO and T80 + EO showed less photobleaching (approximately 4 %) than CEL + M840 and T80 + M840 (approximately 10 %).

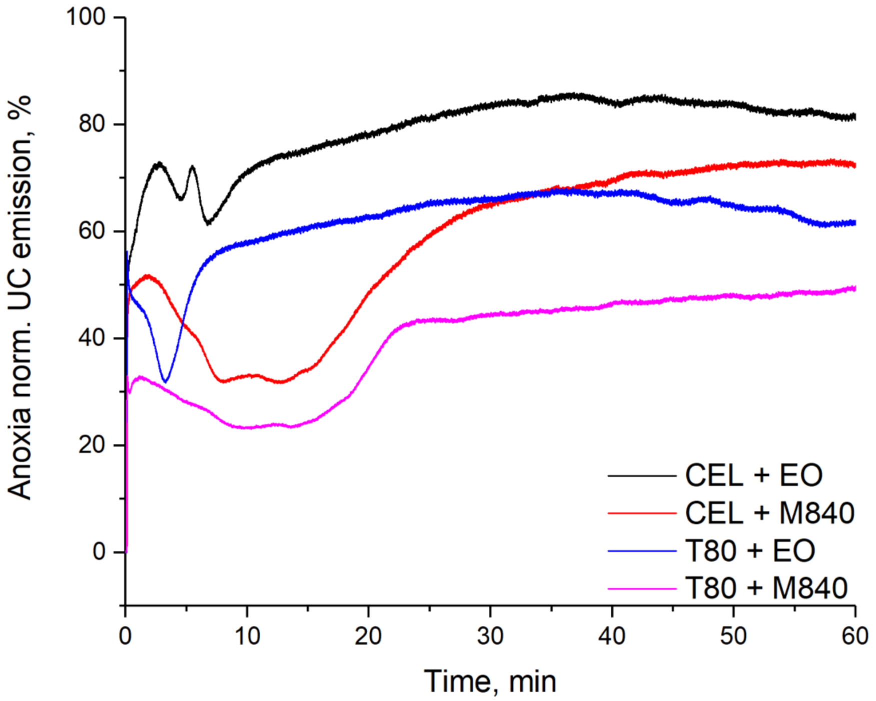

Figure S7. UC emission of the nanocarriers excited at 1000 mW/cm^2 and monitored for 1 hour.

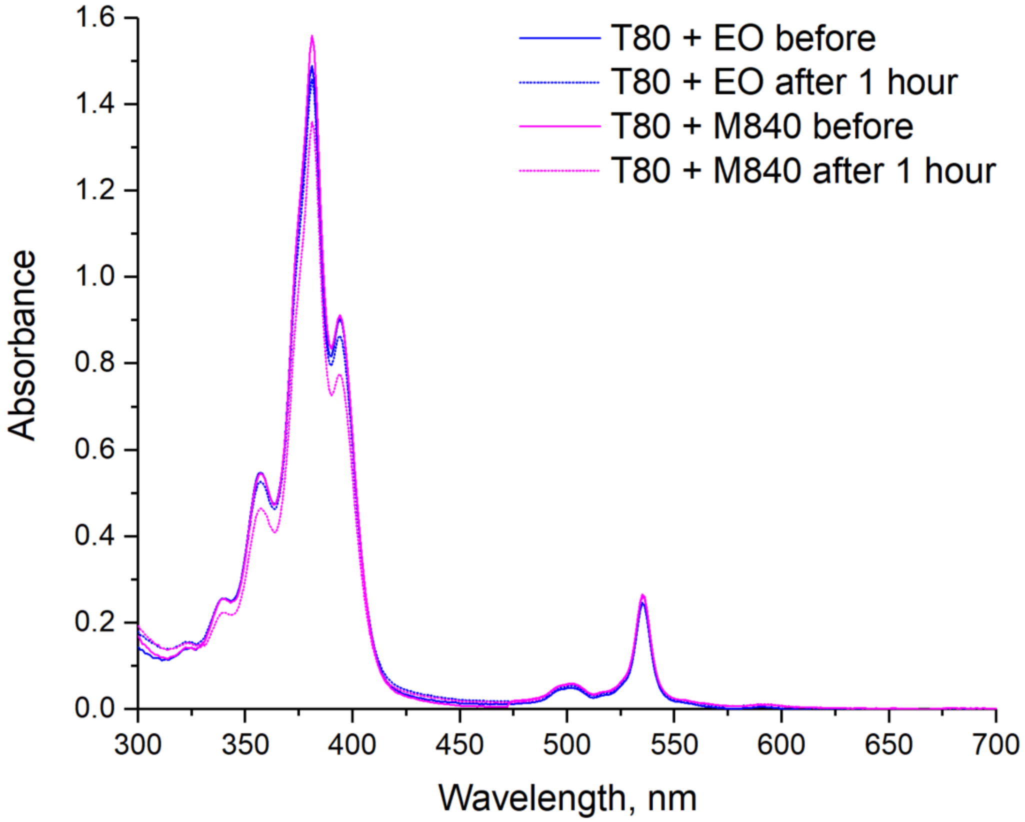

Figure S8. Absorption spectra of T80 + EO and T80 + M840 nanocarriers before and after 1 hour excitation at 1000 mW/cm^2. Photobleaching of DPA is visible in the slight decrease of absorbance between 325 and 425 nm.

Upconversion emission measurements from the microfluidic chip

The upconversion measurements from the microfluidic chip were performed with an excitation power density of 140 mW/cm^2 and the upconverted emission was collected through a 520 nm short-pass filter. The measurement range of the spectrometer was 400–800 nm and thus the emission spectra of 9,10-diphenylanthracene was slightly cut. No changes in the shape of the absorption and emission spectra between the nanocarriers were observed. The emission spectra from T80 + EO nanocarriers under 532 nm excitation is shown in Figure S1.

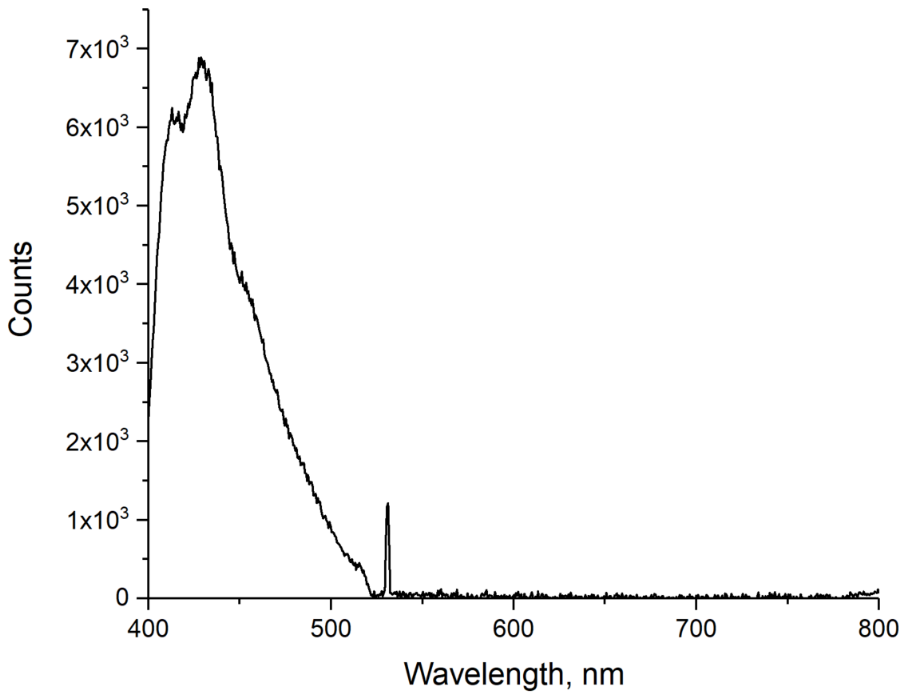

Figure S9. Emission spectrum of T80 + EO nanocarriers under 532 nm excitation detected through a 520 nm short-pass filter.

Upconversion emission intensities at varied oxygen levels normalized by quantum yields

The upconversion intensities measured from the microfluidic chip were normalized by the QY of each nanocarrier at 140 mW/cm^2. These results are shown in Figure S2.

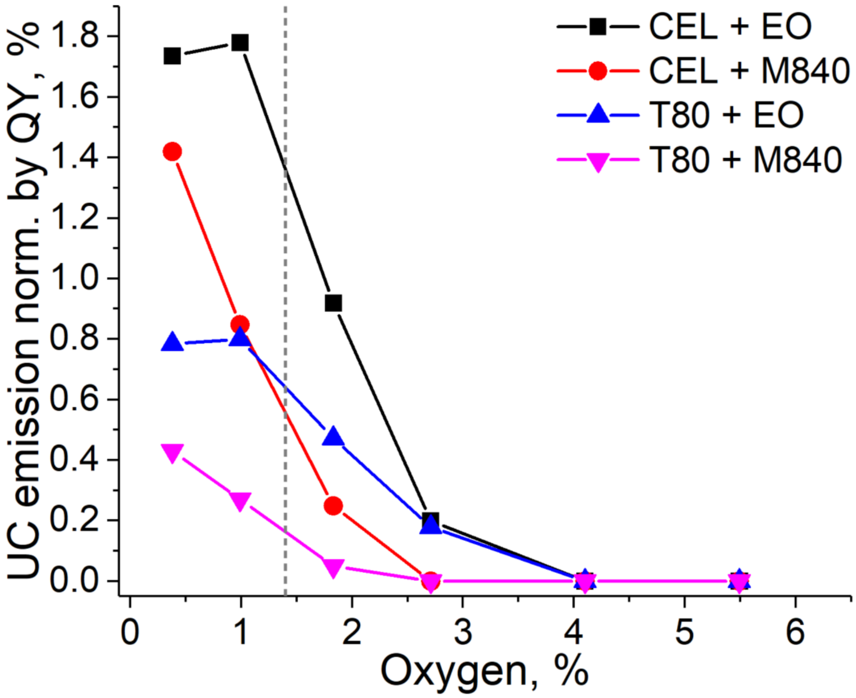

Figure S10. UC emission intensities of the nanocarriers at varied oxygen levels normalized by the QY of respective nanocarrier at 140 mW/cm^2.

References

1 R. F. Kubin and A. N. Fletcher, *J. Lumin.*, 1982, **27**, 455–462.

2 T. N. Singh-Rachford and F. N. Castellano, *Coord. Chem. Rev.*, 2010, **254**, 2560–2573.
